# Editorial

**DOI:** 10.1007/s41125-020-00068-8

**Published:** 2020-09-14

**Authors:** Stefan Kaufmann

**Affiliations:** Centre for Security and Society, Albert-Ludwigs-Universität Freiburg, Werthmannstr. 15, 79098 Freiburg, Germany

The European Journal for Security Research (EJSR) has been successfully worked on and established in the European Community of Security Research by Hans-Jörg Albrecht (Editor-in-Chief) and Christopher Murphy (Managing Editor). Now, there is a change in editorship. After 5 years of responsibility for the EJSR, they leave and special thanks go to both of them for their commitment and the solid basis they have created for the future of the journal. The basic idea with EJSR was to have an interdisciplinary platform encompassing the efforts of humanities and technological sciences in the field of research on societal security. This has proved to be successful in a number of aspects. The range of topics—covering the historical view on environmental safety up to questions about the future regarding military security—indicates the broad horizon of safety and security topics which society members and researchers have been involved with. However, various key issues have become apparent: the security of critical infrastructures, urban security, as well as the increasing technological development, especially the digitization of the security sector. The articles relating to these topics deal with problems of quantitative risk assessment, present engineering conceptualizations of security problems and developments of security solutions, assess the political and societal opportunities and risks of monitoring and control technologies, discuss organizational developments in the security and crisis management of infrastructure operators or with safety authorities and security organizations and examine requirements and transformations of legal structures in view of a dynamization of societal security. They offer a multi-perspective approach allowing for a deep understanding of how—and by means of which strategies and techniques—contemporary societies attempt to establish resilience against various risks, dangers and threats—whether it be terrorism, cyber attacks, organized crime, natural disasters, major accidents or pandemics. Furthermore, the articles raise awareness for the side effects of such security efforts. Some of these side effects with regard to civil rights, social injustice and/or functional paradoxes are not intended, surprising and not predictable but some of them are simply accepted. The journal’s concept reflects a new development of security thinking which tends to consider the categorical separations between “interior” and “exterior,” “civil” and “military,” “safety” and “security,” “disaster” and “crime” as rather obsolete. These separations had determined the institutional arrangement of the safety and security guarantee for a long time. The new security thinking focusses on the basic vulnerability of highly complex and globally interconnected societies. The current coronavirus SARS-CoV-2 pandemic represents in an almost paradigmatic way this problem: The epidemic has been spreading via the global flows of work, trade, tourism and migration. The stopping of these flows involves extensive cascade effects and secondary damage in almost all areas of life—economy, health, academic and social education, civil rights, social relationships and others. The epidemic seems to be the incarnation of the butterfly effect in the chaos theory, and it is the wing beat triggering a disaster and stands for a basic uncertainty and unpredictability of systemic dynamics. Even if the journal currently does not include pandemic topics, this risk profile and questions on how to handle it continue to build the interdisciplinary horizon for this journal—far beyond the classic boundaries of humanities and technological sciences.

In this spirit, the articles in this issue cover a wide range of different topics. The article by Andreas Vasilache analyzes the topicality and the change of meaning of the term “cowardice” as well as the related mobilizing effects in the course of geopolitical tensions and the transformation of international conflicts after the end of the Cold War. These transformations are also examined by Guevara Raul Diaz, who sheds light on the ethical and legal implications when criticizing the state-led war against international terrorism. Christof Nägel and Mark Lutter, too, analyze an anti-liberal effect which counteracts human rights and is not triggered by war against terrorism but by the terror attacks themselves: the spillover effect that negatively shapes public opinion and the attitude toward refugees after the Christmas market attack in Berlin. Kerrin-Sina Arfsten deals with a specific form of prevention of terrorism by exploring the consequences of the appeals made by the Department of Homeland Security to the citizens to practice “terror awareness.” The article by Giacomo Assenza, Andrea Chittaro, Maria Carla De Maggio, Marzia Mastrapasqua and Roberto Setola looks into security awareness programs within the scope of organizational security and discusses various methods for measuring security awareness in order to assess the outcome of such programs. Aishvarya Kumar Jain, Christian Grumber, Patrick Gelhausen, Ivo Häring and Alexander Stolz suggest the use of convolutional neural networks (CNN) for long-term time-series prediction of terrorist event data and demonstrate their applicability in principle as well as their limits and the potential of such an approach. Ivo Häring, Mercy Pfeiffer, Georg Vogelbacher, Alexander Stottmeister, Elena-Maria Restayn, Katharina Ross and Malte von Ramin present a 3D spatial-based method for assessing and handling the hazard of low-cost bomb attacks in urban areas. Jukka Ruohonen raises the issue of European security and its relation to marketization by examining the specific characteristics of cyber security procurement.

